# Expression of Integrin α_v_β_3_ in Gliomas Correlates with Tumor Grade and Is not Restricted to Tumor Vasculature

**DOI:** 10.1111/j.1750-3639.2008.00137.x

**Published:** 2008-07

**Authors:** Oliver Schnell, Bjarne Krebs, Erika Wagner, Alexander Romagna, Ambros J Beer, Stefan J Grau, Niklas Thon, Claudia Goetz, Hans A Kretzschmar, Jörg-Christian Tonn, Roland H Goldbrunner

**Affiliations:** 1 Department of Neurosurgery, Klinikum Grosshadern, Ludwig-Maximilians-Universität MünchenMunich, Germany; 2 Center for Neuropathology and Prion Research, Ludwig-Maximilians-Universität MünchenMunich, Germany; 3 Department of Nuclear Medicine, Klinikum rechts der Isar, Technische Universität MünchenMunich, Germany

**Keywords:** angiogenesis, glioma, integrin α_v_β_3_, integrin immunohistochemistry, tumor vasculature

## Abstract

In malignant gliomas, the integrin adhesion receptors seem to play a key role for invasive growth and angiogenesis. However, there is still a controversy about the expression and the distribution of α_v_β_3_ integrin caused by malignancy. The aim of our study was to assess the extent and pattern of α_v_β_3_ integrin expression within primary glioblastomas (GBMs) compared with low-grade gliomas (LGGs). Tumor samples were immunostained for the detection of α_v_β_3_ integrin and quantified by an imaging software. The expression of α_v_β_3_ was found to be significantly higher in GBMs than in LGGs, whereby focal strong reactivity was restricted to GBMs only. Subsequent analysis revealed that not only endothelial cells but also, to a large extent, glial tumor cells contribute to the overall amount of α_v_β_3_ integrin in the tumors. To further analyze the integrin subunits, Western blots from histologic sections were performed, which demonstrated a significant difference in the expression of the β_3_ integrin subunit between GBMs and LGGs. The presented data lead to new insights in the pattern of α_v_β_3_ integrin in gliomas and are of relevance for the inhibition of α_v_β_3_ integrin with specific RGD peptides and interfering drugs to reduce angiogenesis and tumor growth.

## INTRODUCTION

Gliomas are the most common brain-derived neoplasms with glioblastoma (GBM) multiforme being the most frequent and also the most malignant subtype. Marked tumor cell proliferation, rapid invasion into the surrounding brain tissue and intense microvascular angiogenesis are the biologic hallmarks of malignant gliomas (25, 26, 34). They depend on the complex interactions of tumor cells with the extracellular matrix (ECM) and neighboring endothelial cells as well as various other cell types (14, 40). These mechanisms include signaling via integrins, which are involved in adhesive properties of proliferating cells and play fundamental roles in the regulation of migration and invasion of tumor cells as well as of neoangiogenesis (23, 36). The integrin family consists of 24 different heterodimeric receptors of two transmembrane subunits of which the 18α and 8β subunits are known at present (5, 23, 41). In contrast to many other cell-surface receptors, integrins are not only able to transduce signals to the interior of the cells (“outside-in” signaling) but can also transmit information about the activational state of the cells to their microenvironment (“inside-out” signaling) (13, 41).

The integrins α_v_β_3_ and α_v_β_5_ have been demonstrated to be necessary for tumor-induced angiogenesis in a variety of tumors, particularly malignant gliomas via basic fibroblast growth factor (bFGF) and tumor necrosis factor α (TNF-α) (9, 10, 15). Indeed, integrin α_v_β_3_ has been found to be located in small blood vessels in GBMs, where it is thought to promote the extensive tumor progression, while in the tissue of the normal brain, it is barely detectable (16). Interestingly, immunohistochemical studies of autopsy material from patients with brain tumors revealed that the expression of integrin α_v_β_3_ might not be restricted only to proliferating endothelial cells (32). Integrin α_v_β_3_ was also detected and colocalized with matrix metalloproteinase 2 (MMP-2) at the surface of invasive tumor cells (6, 11). Yet, the level of α_v_β_3_ integrin expression and its cellular distribution caused by malignancy has not been expounded up to now. This has become even more important as α_v_β_3_ integrin antagonists have reached phase I/IIa clinical trials in patients with malignant gliomas, where they are used in combination with temozolomide as an antiangiogenic therapy. The current study was undertaken to elucidate the expression level and pattern of the integrin α_v_β_3_ in high- and low-grade gliomas (LGGs) by analysis of snap-frozen tumor samples.

## MATERIALS AND METHODS

### Patients and tissue samples

The brain tissues were obtained from 20 primary GBMs, corresponding to the World Health Organization (WHO) grade IV, and five diffuse astrocytomas, corresponding to WHO grade II. Written informed consent was obtained from all patients for the scientific use of tumor tissue. The histologic diagnosis was made on paraffin sections according to the WHO criteria (25). For the immunohistochemical analysis of α_v_β_3_ integrin expression (GBM: n = 12; LGG: n = 4; histologically suitable quality) and Western blot analysis (GBM: n = 20; LGG: n = 5), corresponding tissue samples were snap frozen in liquid nitrogen and processed as described below. Additionally, samples of five different solid peripheral tumors (malignant melanoma, neurofibroma, skin cancer metastases, malignant fibrillary histiocytoma and sarcoma; n = 1 each) were processed (kindly provided by the Department of Pathology, Technical University of Munich, Munich, Germany).

### Histologic and immunohistochemical staining

#### Paraffin sections

For the histologic tumor grading, tissues from all patients were fixed for at least 24 h in phosphate-buffered formalin (4%, pH 7.3), dehydrated in graded ethanol followed by xylol and embedded in hot paraffin. Sections (6 µm) were cut from a cold paraffin block and mounted on slides. After drying and deparaffination in graded alcohol, routinely Hematoxilin & Eosin (H&E), Elastica van Gieson and Gomorri stains were performed. Additional immunohistochemical investigations included the proliferation index by staining proliferation marker MIB-1 and glial fibrillary acid protein (GFAP) according to standard procedures (antibodies were obtained from DakoCytomation, Hamburg, Germany).

#### Cryosections

Cryosections of 6-µm thickness (GBM: n = 12; LGG: n = 4; histologically suitable quality) were mounted on glass slides (Superfrost Plus, Menzel, Braunschweig, Germany) and postfixed with 4% paraformaldehyde and acetone (each 10 minutes at 4°C). After a short equilibration in phosphate-buffered saline (pH 7.3) over 10 minutes at room temperature, immunohistochemical stains were performed simultaneously and automatically to reach a maximum of accuracy (BenchMark® and software NexES v9.20, Ventana, Strasbourg, France). As primary antibodies, we used either polyclonal anti-GFAP (1:1600, DakoCytomation, Hamburg, Germany), monoclonal anti-CD31 (1:50, DakoCytomation, Hamburg, Germany) or monoclonal anti-α_v_β_3_ integrin (1:100, clone LM609, Chemicon, Temecula, CA, USA) diluted in antibody diluent (Antibody Diluent, Ventana, Strasbourg, France). The primary antibody was incubated over 32 minutes at 37°C (software: “option 1 per hand”). Respective application of polyclonal biotinylated secondary antibodies (1:150, DakoCytomation, Hamburg, Germany, secondary antibody incubation 32 minutes at 37°C, software: “option 2 by dispenser”) was followed by alkaline phosphatase conjugated streptavidin (enhanced SA-AP over 12 minutes, Ventana, Strasbourg, France). Visualization was achieved by the generation of Fast Red over 20 minutes at room temperature as prescribed by the manufacturer (enhancer/naphthol 4 minutes, Fast Red A 8 minutes, Fast Red B 8 minutes, Ventana, Strasbourg, France). Subsequently, the slides were counterstained with hematoxylin (hematoxylin 4 minutes, bluing reagent 4 minutes) and 4′-6-Diamidino-2-phenylindole (DAPI). The slides were dehydrated by an ascending ethanol sequence (50%, 70%, 98%, 100%) and xylene, and finally covered.

All immunohistochemical staining procedures and antibody concentrations were adjusted to avoid overstaining (saturation), understaining (beyond detection limit) or non-specific background artifacts. In detail, for staining α_v_β_3_ integrin, series of dilutions of the secondary antibody (previous incubation without primary antibody) were performed on positive control (xenotransplanted human M21 melanoma cells expressing α_v_β_3_, see below) to determine the highest concentration where no background artifacts or non-specific binding occurred (= maximum sensitivity of secondary detection). Then, based on this setting, series of the primary antibody concentration were tested on the positive control. As staining intensity demonstrates a saturation effect, the concentration developing a moderate staining intensity (about half of the maximum intensity of a blank sample) was further used. At this concentration, no non-specific background staining was detectable.

### Murine tumor models

Xenotransplanted human melanoma cells (M21) expressing α_v_β_3_ integrin (12, 19) served as a positive immunohistochemical control. Cell culture conditions for M21 were set up as described elsewhere (20). The experimental protocol involving animals was approved by the Committee of Veterinarian Medicine of the State of Bavaria; the handling of animals was performed according to the standards set by the Committee of Veterinarian Medicine.

To obtain the tumor tissue, the mice were injected 1.5 × 10^6^ M21 subcutaneously, which led to the formation of tumors with a diameter of approximately 8 mm within 8 weeks. Four weeks after inoculation, the tumors were dissected, cut and frozen for further processing (20).

### Analysis of immunohistochemical staining

The immunohistochemical detection of α_v_β_3_ integrin was determined by a special imaging software, which was calibrated for this purpose. Within a selected area, it measured (i) the mean intensity of the desired immunohistochemical staining and (ii) the immunohistochemically positive fraction at a desired immunohistochemical staining intensity (software written by the author B. Krebs) (33).

In detail, all sections stained in parallel were scanned through an upright microscope (Axiovert 200M and Axiocam MRc5, Carl Zeiss AG, Jena, Germany), keeping the settings including light intensity constant. In the digitized pictures (983 × 737 µm), the area of tissue was selected by excluding possible empty areas of glass light only. Consequently, the measurement was related to tissue only. Then, the selected area was scanned for immunohistochemical detection. Thereby, the software mathematically extracted the component of the desired immunohistochemical staining per pixel (Fast Red maximum emission: 580 nm) and determined its intensity. From this data, the mean intensity of the immunohistochemical staining was calculated. For calibrating the intensity measurement, a sample stained in parallel of xenotransplanted human M21 melanoma cells expressing α_v_β_3_ was used. This sample acted as a positive control (12, 19) and was set to 100%. The tissue that was counterstained only served as a negative control (set to 0%). The procedure resulted in (i) the relative level of α_v_β_3_ integrin expression in the respective sample (= mean immunohistochemical staining intensity). In addition, to evaluate the distribution pattern of the immunohistochemical staining intensity within the selected area, the fraction of immunohistochemically positive area at a desired intensity level was calculated. In this study, three different levels of staining intensity defined as mild, moderate or strong (about 25%, 50% and 75% of positive control) were investigated. This resulted in (ii) a quantification based on the desired levels of staining intensity (= fraction at different intensity levels).

### Western blot

Parallel to the immunohistochemical stainings, Western blotting was performed from the frozen sections of the brain tumor biopsies as described elsewhere (27). In brief, frozen brain sections were cut in 6-µm sections and mounted on glass slides (same as for immunohistochemical stainings). Afterwards, the sections were overlaid with section lysis buffer [2% sodium dodecyl sulfate (SDS), 0.05 M dithiothreiotol, 10% glycerol, 1 mm EDTA, 16 mm Tris-HCl, pH 7.2] using a total volume of 20 µL per 10 mm^2^ of tissue. The solubilized tissue was collected, boiled for 10 minutes and subjected to sodium dodecyl sulfate polyacrylamide gel electrophoresis. The very small amount of tissue used in this study was not suitable for standard methods of protein measurement (eg, Bradford assay). Therefore, a protein loading control was determined separately (β-actin, see beyond). Proteins were electrophoretically separated (New Page Gel 12%, Invitrogen, Karlsruhe, Germany) with 100 V over 24 minutes in running buffer [0.1% SDS, 1 mm EDTA, 15 mm 3-(N-morpholino) propansulphonic acid (MOPS), 50 mmol Tris-HCl, pH 7.2]. After that, the proteins were transferred to a polyvinyldifluoride (PVDF) membrane (0.45 µm, Immobilon, Millipore, Eschborn, Germany) by a semidry blotting technique with freshly prepared blotting buffer (20% methanol, 192 mmol glycin, 25 mmol Tris-base, pH 8.3). Next, the PVDF membranes were blocked in blocking buffer (0.05% w/v casein, 0.5% v/v Tween in phosphate buffer saline, pH 7.4) over 1 h at room temperature. The primary antibodies against integrin subunits (polyclonal rabbit anti-integrin α_v_ subunit AB1930 and polyclonal rabbit anti-integrin β_3_ subunit AB1932; Chemicon, Temecula, CA, USA) were diluted in the respective blocking buffer (1:1000) and incubated overnight at 4°C. After a washing step, bound antibodies were labeled using a secondary antibody coupled to alkaline phosphatase (1:2000 polyclonal goat anti-mouse and anti-rabbit immunoglobulin/AP, DakoCytomation, Hamburg, Germany) over 45 minutes. Visualization followed a second washing step using chromogen nitroblue tetrazolium (NBT) salt and 5-bromo-4-chloro-3-indulylphosphate (BCIP) according to standard protocols (28). The wet blots were scanned using a high-resolution flatbed scanner before reprobing. For the detection of the endothelial component, the blots were reprobed with anti-CD31 (CD31); for the assessment of the amount of tissue, Western blots were reprobed with anti-β-actin (Actin I-19, Santa Cruz Biotechnology, Heidelberg, Germany) as the ubiquitous component of the cytoskeleton (21). Densitometric measurements were performed with the software TotalLab V 2.01 (Nonlinear Dynamics, Newcastle upon Tyne, UK) by using series of dilution for calibration of the concentration and a protein ladder (Invitrogen, Karlsruhe, Germany) for the calibration of the molecular weights.

### PCR

For the analysis of the mRNA expression of integrin subunits α_v_ and β_3_, equal amounts of tumor tissue from GBMs (n = 5) and LGG (n = 3) patients were homogenized and suspended in TRIZOL® reagent (Invitrogen, Karlsruhe, Germany). RNA was isolated according to the manufacturer's instructions (Invitrogen, Karlsruhe, Germany). The first-strand cDNA synthesis was done by MMLV-RT (New England Biolabs, Frankfurt, Germany). Primers were designed according to the sequences of ITAV [accession number NM_002210; forward primer (2692–2716): 5′-CTATGAGCTGAGAAACAATGGTCC-3′; reverse primer (3351–3373): 5′-GCTGCTCCCTTTCTTGTTCTTC-3′] for integrin subunit α_v_ and ITB3 [accession number NM_00212; forward primer (1859–1881): 5′-CTGTATCCAGCCGGGCTCCTATG-3′; reverse primer (2346–2369): 5′-GCCCCGGTACGTGATATT GGTGAA-3′] for integrin subunit β_3_. The analysis and comparison of the sequences were performed with the DNAstar software (GATC Biotech, Konstanz, Germany). All primers were blasted against the www-databases. PCR for amplifying total cDNA as the template was performed for up to 30 cycles with denaturation for 30 s at 95°C, annealing for 45 s at 64°C and elongation for 60 s at 72°C with the Advantage™ 2 PCR Enzyme Systems (Takara, Otsu, Japan).

### Statistics

Staining intensities and densities of immunopositive structures as well as densitometric measurements of the Western blots were tested for normal distribution and groups were compared by Student's *t*-test for unpaired values and unequal variances. All values were given with standard deviation.

## RESULTS

### Two types of patterns of α_v_β_3_ and CD31 expression

**Figure 1:**
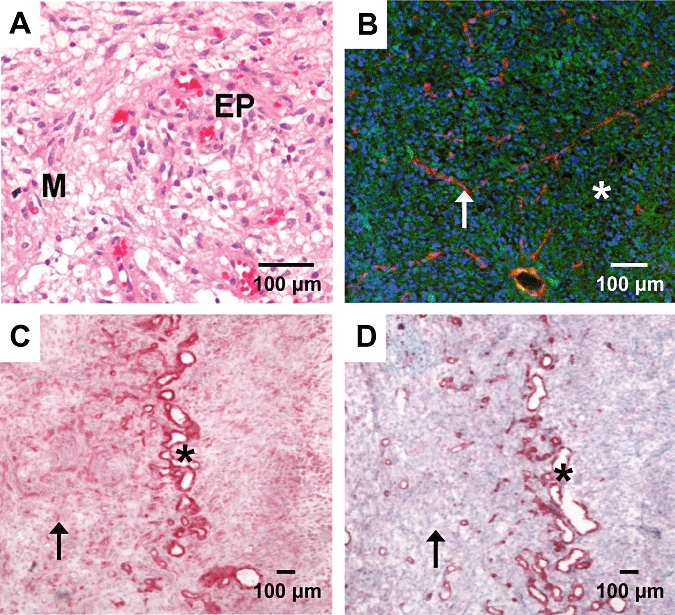
Hematoxilin & Eosin (H&E) (**A**) and immunostain of integrin α_v_β_3_ (**C**), CD31 (**D**) and fluorescent overlay (**B**) in samples of glioblastoma. Standard H&E staining (**A**) shows typical morphology of a malignant glioma. Immunohistochemical staining of α_v_β_3_ (**C**) is intense in vascular structures (asterisk) as confirmed by a consecutive section stained for the endothelial cell marker CD31 (**D**). Rather, α_v_β_3_ is clearly detectable throughout the whole section as expressed by the glial tumor cells (arrow). A fluorescent overlay picture of another area with dense tumor growth (**B**) demonstrates that the integrin α_v_β_3_ (green) is not restricted to vascular structures (red, asterisk) but shows a ubiquitous distribution pattern (cell nuclei blue). Scale bars: 100 µm. Abbreviations: EP = endothelial proliferation; M = mitosis.

**Figure 2:**
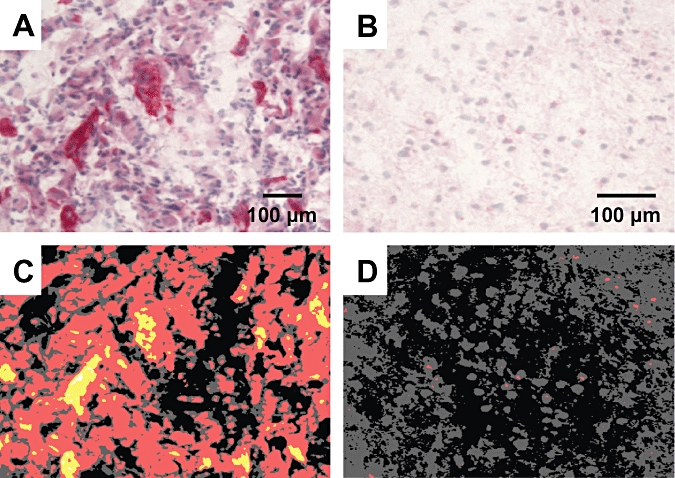
Detection of α_v_β_3_ integrin in malignant (**A**) and a low-grade (**B**) glioma and analysis by an imaging software (**C**,**D**). The malignant glioma (**A**) exhibit a much higher expression of α_v_β_3_ integrin (red) than the low-grade glioma (**B**), where only very few glial cells are immunopositive for the integrin. The detection of the staining intensity in the malignant glioma shows a large overall baseline (red) expression with certain strong (yellow) or even intense (white) expression in microvascular structures (**C**). In contrast, only some red dots can be detected in the low-grade glioma (**D**). Scale bars: 100 µm

Immunohistochemical staining of α_v_β_3_ integrin was investigated in sections of GBMs (n = 12), diffuse low-grade astrocytomas (n = 4) as well as non-CNS tumors (n = 5). The expression of α_v_β_3_ integrin was found in the microvessels as well as in the glial tumor cells. The majority of α_v_β_3_ expression in glial tumors was located in samples from GBMs, which are characterized by extensive proliferation of pleomorphic glial cells accompanied by microvascular proliferates with branching glomeruloid vessels. A sample of an HE stain is given in Figure 1A. Figure 1C,D demonstrates the immunohistochemical stainings of an area with vital tumor (left) and a hypercellular zone (mid) surrounding the necrotic areas (right). The staining for α_v_β_3_ integrin, shown in Figure 1C, demonstrates a high endothelium-associated expression in this hypercellular zone. This expression is almost identical to the staining of endothelial marker CD31, as demonstrated in Figure 1D (microvascular association). Nevertheless, numerous tumor cells, especially enlarged giant pleomorphic astrocytes, show a strong immunoreactivity for the α_v_β_3_ integrin, too. In other samples or areas with dense tumor growth, the expression of α_v_β_3_ integrin seems to be associated to glial tumor cells as well. A typical sample is demonstrated by an overlay of fluorescent stainings of α_v_β_3_ integrin and CD31 in Figure 1B. The small branched proliferating microvessels marked by CD31 in red are only partially colocalized with the expression of the α_v_β_3_ integrin in green, which is predominantly located in the glial tumor cells (glial association). The nuclei are counterstained in blue. In contrast to the GBMs, a low expression of α_v_β_3_ integrin is found in WHO grade II diffuse astrocytomas, where the staining was more diffuse and only barely associated with the microvessels (Figure 2B).

### α_v_β_3_ Protein expression is associated with malignancy

**Figure 3:**
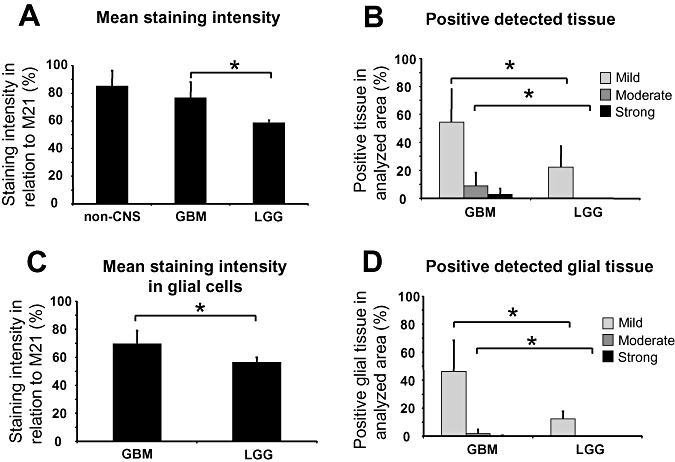
*Statistical analysis of the α_v_β_3_ integrin immunohistochemistry in patients with glioblastomas (GBMs) and low-grade gliomas (LGGs) and non-CNS tumors (non-CNS)*. **A.** Mean staining intensity of non-CNS tumors, GBMs and LGGs. **B.** Fraction of mild, moderate and strong stained tissue in GBMs and LGGs. **C.** Mean staining intensity of glial tissue only in GBMs and LGGs. **D.** Fraction of mild, moderate and strong stained tissue of glial tissue only in GBMs and LGGs. Significant differences (*P* < 0.05, Student's *t*-test) are indicated with an asterisk.

The different samples were simultaneously stained for α_v_β_3_ integrin (Figure 2A,B) and analyzed via an imaging software (Figure 2C,D). The measurements were calibrated to xenotransplanted human melanoma cells (M21) expressing α_v_β_3_. These acted as a positive control and were set to 100% integrin α_v_β_3_ expression. The overall mean staining intensity of α_v_β_3_ integrin in GBMs reached 76.53 ± 12.08%, which was significantly higher than in LGGs with 58.43 ± 2.30% (*P* < 0.05, Figure 3A). In comparison, the samples of the non-CNS tumors reached 85.18 ± 11.95%, (*P* < 0.05).

To investigate the expression pattern in more detail, the fraction of immunohistochemically positive structures (density) were calculated for mild, moderate and strong positive detection of α_v_β_3_ integrin (demonstrated by different colors in Figure 2C,D). In the samples of GBMs, 54.62 ± 23.52% of all tissue were found at least mildly stained, whereas the samples of LGGs reached only 22.20 ± 14.99% (*P* < 0.05, Student's *t*-test; Figure 3B). The moderate staining of α_v_β_3_ integrin was nearly exclusively associated with malignancy, with 9.07 ± 9.25% in GBMs and 0.06 ± 0.07% in low-grade astrocytomas (*P* < 0.05, Student's *t*-test). The strong staining intensities were restricted to GBMs only (2.83 ± 4.08%; Figure 3B). Therefore, the average α_v_β_3_ integrin expression in GBMs exceeds α_v_β_3_ integrin expression in LGGs by far. However, different GBMs show a very heterogeneous expression of α_v_β_3_ integrin, which ranges from slightly to very strong integrin expression, similar to high expressing non-CNS tumors.

### Glioma cells substantially contribute to α_v_β_3_ integrin expression

To access the glial cell specific α_v_β_3_ integrin expression within the tumor, small areas were selected and analyzed where no endothelial structures were present. These areas showed once more a significantly higher mean staining intensity in GBMs (69.47 ± 9.49%) than in LGGs (56.41 ± 6.83%; *P* < 0.05). The density of immunohistochemically positive tumor cells was measured in GBMs (mild: 46.31 ± 22.23%, moderate: 2.02 ± 2.88%, strong: 0.23 ± 0.49%) and LGGs (mild: 12.31 ± 5.46%, moderate: 0.01 ± 0.01%, strong: 0%), which were significantly different (*P* < 0.05), although the smaller areas might increase the sampling error. The latter findings indicate that the tumor cells contribute substantially to the α_v_β_3_ integrin expression.

In conclusion, GBMs demonstrated a higher expression of α_v_β_3_ integrin than low-grade astrocytomas that was not only caused by a high focal reactivity in proliferating microvessels but also by stronger expression in glial tumor cells. The pattern of α_v_β_3_ integrin expression depended also on the subtype of tumor. In malignant gliomas, more than three quarters of the overall integrin expression (about 85%) is derived from glial tumor cells.

### β_3_ Integrin subunit expression shows an essential difference between GBMs and LGGs in Western blot analysis

**Figure 4:**
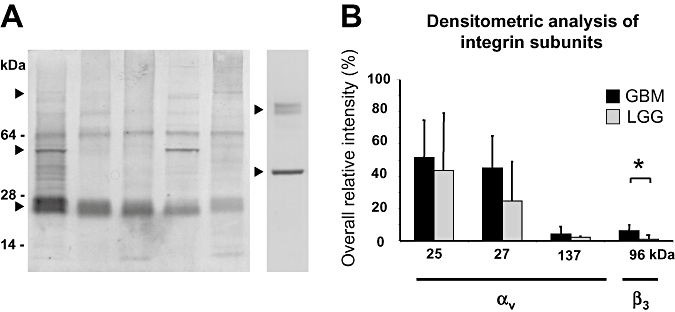
Western blot analysis of subunits and degradation products of the α_v_β_3_ integrin. The Western blot analysis of five different glioblastoma (GBM) tissue samples (**A**, left lanes) and densitometric analysis of integrin subunits (**B**). In all glial tumors, degradation products of the α_v_ subunit (α_v_ light chains) were detected as a double band at 25/27 kDa (▸). Expression was constantly present but heterogeneous between different GBMs. The β_3_ subunit was detected at about 96 kDa (▸). Degradation products of α_v_ and β_3_ subunit were found at 55 kDa (▸). The detection of CD31 at about 80 kDa (right ▸) and β-actin at about 42 kDa (right ▸) is additionally demonstrated in the lane on the right. **B.** Densitometric analysis revealed significant differences for the β_3_ subunit between malignant gliomas and low-grade gliomas (LGGs) (*P* < 0.05; Student's *t*-test; indicated with an asterisk). The α_v_ subunit (about 137 kDa) and its degradation products (α_v_ light chains, 25/27 kDa) showed no significant differences between high- and low-grade gliomas.

To investigate the immunohistochemical expression of integrin α_v_β_3_ expression in more detail, we performed Western blot analysis from frozen brain tumor samples as described in the methods. Each lane was loaded with an equal tissue volume of 30 nL (5 mm^2^ of tissue). The Western blots were incubated with subunit specific antibody for β_3_ chain and α_v_ chains. Thereby, the integrin subunits demonstrate degradation to potential cleavage products. The β_3_ chains were detected as weak bands at a molecular weight of about 96 kDa accompanied by their major degradation products at about 64 and 52 kDa. The heavy α_v_ chains were detectable at 137 kDa. Further on, strong double bands were detectable at about 25 and 27 kDa, which correspond to major degradation products α_v_ light chains. The full lengths of the α_v_ light chains are barely detectable at about 52 kDa. Figure 4A shows the detection of integrin subunits from five different GBM patients (left five lanes; the one lane on the right demonstrates CD31 and β-actin). The group of GBMs demonstrate a heterogenous pattern of β_3_ chains, whereas the portions of α_v_ light chains were strongly present in all samples. The expression in low-grade astrocytomas was lower, especially the β_3_ chains, which were barely detectable.

The densitometric analysis (Figure 4B) of the samples, calibrated by the dilution series of a reference probe, revealed an overall density of β_3_ chain at 96 kDa and α_v_ light chains at 25 and 27 kDa of 102.8 ± 43.0 in GBMs (n = 20) and 69.1 ± 30.9 in low-grade astrocytomas (n = 5), which corroborates the immunohistochemical measurements (but is not statistically significant). By comparing the β_3_ subunit at 96 kDa, we found a highly significantly elevated expression in GBMs with 6.18 ± 4.59 compared with LGGs with 1.07 ± 0.549 (*P* < 0.05; Student's *t*-test, Table 1). The expressions of α_v_ light chains at 25 and 27 kDa were not significantly different in GBMs (51.6 ± 23.0 and 45.0 ± 19.9 respectively) and in low-grade astrocytomas (43.4 ± 35.6 and 24.6 ± 24.6, respectively).

**Table 1:** Densitometric analysis of integrin subunits α_v_ and β_3_ and microvascularization in glioblastomas (GBMs), low-grade gliomas (LGGs) and non-CNS tumors (non-CNS). Overall density of integrin subunits α_v_ (25/27 kDa) and β_3_ (96 kDa) as well as the endothelial cells (CD31; 80 kDa) of the tumors was assessed by the Western blot analysis and calibrated by a dilution series of a reference probe. In GBMs, the β_3_ subunit at 96 kDa showed a significantly elevated expression compared with LGGs (*P* < 0.05; Student's *t*-test). The amount of CD31 demonstrated quite different values in GBMs and in LGGs, indicating microvascular proliferation.

	α_v_ Light chains					
		
	25 kDa	27 kDa	β_3_ Subunit 96 kDa	α_v_ + β_3_ Subunit 25 + 27 + 96 kDa	CD31 80 kDa	β-Actin 40 kDa
GBM	51.6 ± 23.0	45.0 ± 19.9	6.18 ± 4.59	102.8 ± 43.0	25.2 ± 12.9	36.8 ± 22.7
LGG	43.4 ± 35.6	24.6 ± 24.6	1.07 ± 0.549	69.1 ± 30.9	4.9 ± 2.2	30.1 ± 13.2
Non-CNS	36.2 ± 29.0	70.0 ± 36.1	8.1 ± 6.2	105.3 ± 58.0	34.6 ± 18.4	52.5 ± 41.0

Further on, microvascularization was assessed by Western blot analysis using the endothelial cell marker CD31, which was detected at 80 kDa (Figure 4A, right lane). Densitometric analysis demonstrated that samples of GBMs had significantly higher staining intensities (25.2 ± 12.9) than LGGs (4.9 ± 2.2, *P* < 0.05). The ratio of the overall integrin α_v_β_3_ detection (sum GBMs 102.8 ± 43.0, sum LGGs 69.1 ± 30.9) to the amount of CD31 demonstrated quite different values of 4.1 in GBMs and 14.1 in LGGs, which indicates that the detection of CD31 positive endothelial cells overcomes the higher expression of α_v_β_3_ integrin in GBMs (Table 1). Consequently, the numerous proliferating endothelial cells do not contribute at all to the α_v_β_3_ integrin expression.

### Integrin subunits α_v_ mRNA and β_3_ mRNA both found in GBMs and low-grade astrocytomas

**Figure 5:**
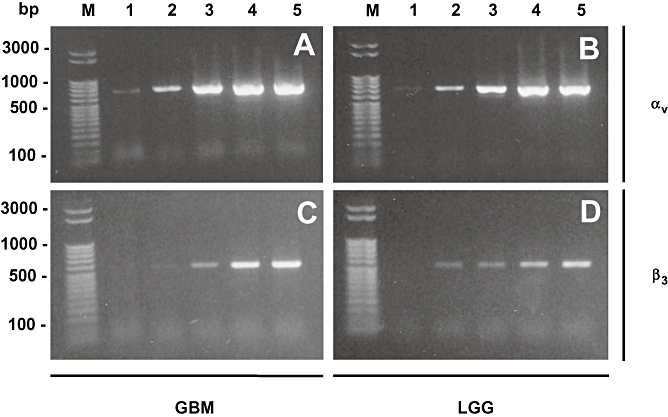
PCR analysis of α_v_β_3_ integrin subunits. PCR analysis of α_v_β_3_ integrin subunits α_v_ (**A**,**B**) and β_3_(**C**,**D**) of a glioblastoma (GBM; **A**,**C**) and low-grade glioma (LGG; **B**,**D**) after different recombination cycles. Lane 1: 24 cycles, lane 2: 27 cycles, lane 3: 30 cycles, lane 4: 33 cycles and lane 5: 36 recombination cycles. Standard PCR revealed that both subunits α_v_ (690 bp) and β_3_ (510 bp) are synthesized in high as well as low-grade gliomas indicating that both tumor entities are able to express a functionally active α_v_β_3_ integrin receptor. Marker (M) was a 50 bp ladder (Novagen, Perfect DNA ladder).

As immunohistochemical stainings and Western blot analysis revealed an α_v_β_3_ integrin expression in malignant GBMs and in a low level in low-grade astrocytomas, we investigated the expression on mRNA level. Cryoconserved tissue of low- and high-grade gliomas was subjected to RT-PCR as described in the materials. Figure 5 demonstrates the PCR product after 24 to 36 recombination cycles for the subunits α_v_ and β_3_ in a sample of a malignant GBM and a low-grade astrocytoma. The mRNA of subunit α_v_ was strongly amplified in both high- and low-grade gliomas. The mRNA of the β_3_ subunit was also present in both tumor entities but amplified on a lower level. Therefore, on the mRNA level, both malignant GBM and low-grade astrocytoma were able to express active α_v_β_3_ integrin, as suggested by the previous immunohistochemistry and Western blotting, which corroborates the previous results of immunohistochemistry and Western blotting.

## DISCUSSION

Integrins are adhesion receptors that mediate cell–cell as well as cell–ECM interactions associated with tumor growth and angiogenesis in malignant gliomas (5, 23, 31, 39). However, there is still a controversy about the extent and the distribution of α_v_β_3_ integrin expression caused by malignancy and the cell type of origin in gliomas. In literature, 30% of glioma cells were described positive for α_v_β_3_ integrin in anaplastic astrocytomas and GBMs, whereas LGGs did not express detectable amounts of this integrin (16–18, 38). *In situ* studies for the two different integrin subunits α_v_ and β_3_ revealed that the expression of integrin α_v_β_3_ was focused more on small blood vessels of GBMs (16, 38). One other group detected individual glial tumor cells of GBMs to be positive for the subunits α_v_ and β_3_(32). Only recently, Bello *et al* reported a more prominent expression of integrin α_v_β_3_ on glioma cells but without providing a detailed expression pattern (5). Yet, this is a matter of major interest for therapeutic delivery of integrin α_v_β_3_ interfering agents to reduce tumor angiogenesis and growth (7, 9, 29, 38). The efficacy of this approach is clearly dependent on the intratumoral extent and distribution of α_v_β_3_ integrin, as it has to be supposed that mainly the endothelial cell bound α_v_β_3_ integrin portion is reached by intravenous application.

For this reasons, the extent, distribution and pattern of integrin α_v_β_3_ expression as well as its correlation to tumor grade were investigated. The computed immunohistochemistry and calibrated staining analysis provided high accuracy and the possibility to bypass the very crucial point of visual quantification of immunohistochemical staining results. In GBMs, a high expression of α_v_β_3_ integrin was found not only in the endothelial cells of proliferating microvessels but also in tumor cells. The great variability of the expression might, in part, explain why the studies that have been carried out so far showed controversial results according to the amount of α_v_β_3_ integrin and its cellular and regional distribution within the tumor. While previous reports focused more on the impact of integrin α_v_β_3_ on angiogenesis (5, 16), we demonstrated that glial tumor cells themselves contribute significantly, accounting for approximately 85% of the overall expression of α_v_β_3_ integrin. In contradiction to former studies, we were also able to detect integrin α_v_β_3_ expression even in LGGs (18), but on a significantly lower level and predominantly on tumor cells, as there is a lower vascularization.

Interestingly, our miniaturized Western blot analysis revealed a significant difference only for the expression of the β_3_ subunit between GBMs and LGGs, whereas the α_v_ subunit and its degradation products (12) showed no significant difference between these tumor entities. This might be caused by the fact that the integrin α_v_ subunit is also needed with the β_5_ subunit to form the α_v_β_5_ integrin, which is also abundant in highly vascularized malignant gliomas (5). Moreover, there are other β subunits (β_1_, β_6_, β_8_) that heterodimerize with the α_v_ subunit, whereas the β_3_ subunit in brain tumors only partners with α_v_ and with α_IIb_ in thrombocytes (5, 18, 23, 24, 32). Therefore, the endothelial expression of α_v_β_3_ integrin seems to be characteristic for malignant microvessel transformation, as it has already been described that the β_3_ subunit expression in GBMs is most prominent in endothelial and perivascular cells associated with tumor angiogenesis (5, 16, 18). Moreover, integrin α_v_β_3_ has already been detected in colocalization with MMP-2, where it was found to correlate with the dedifferentiation and invasive behavior of invasive endothelial cells as well as tumor cells (5, 8, 9, 11, 37). As MMP-2 is an important metalloproteinase for the invasion and dissemination of tumor cells, its functional relationship to the α_v_β_3_ integrin expression might play a key role in tumor progression (30). However, recent studies on genetically altered mouse models raise the question whether the integrin α_v_β_3_ is truly proangiogenic and necessary for tumor angiogenesis, as mice lacking either one of these subunits are viable and fertile and show extensive vascularization (1, 22, 35).

Despite these still unanswered questions, antiangiogenic therapy with integrin α_v_β_3_ antagonists has reached clinical phase I/IIa trials for the patients with malignant gliomas. Therefore, minute detection and quantification of α_v_β_3_ integrin expression could be a prerequisite for the selection of patients suitable for this kind of additional therapy to analyze the potential relationship between α_v_β_3_ expression and antitumor activity. In the context of a modern personalized cancer therapy, the benefits should be calculated for each individual patient, especially, as GBMs show a very heterogenous expression of α_v_β_3_ integrin, which ranges from only mild to very strong. This might also include the noninvasive detection of α_v_β_3_ expression in gliomas through molecular imaging methods (2–4, 19, 20, 30). Thus, future studies will have to confirm that either tumor angiogenesis or higher malignancy in gliomas in fact correlate with the amount of integrin α_v_β_3_ expression. This could also lead to new diagnostic and therapeutic approaches for integrin-interfering agents.
